# Retrospective assessment of HDR brachytherapy dose calculation methods in locally advanced cervical cancer patients: AcurosBV vs. AAPM TG43 formalism

**DOI:** 10.1002/acm2.14549

**Published:** 2024-10-09

**Authors:** Billie Ann Radcliffe, Yongbok Kim, Julie Raffi, Diandra N. Ayala‐Peacock, Sarah J. Stephens, Junzo Chino, Sheridan Meltsner, Oana Craciunescu

**Affiliations:** ^1^ Department of Radiation Oncology Cape Fear Valley Health Fayetteville North Carolina USA; ^2^ Department of Radiation Oncology Duke University Medical Center Durham North Carolina USA

**Keywords:** Acuros, brachytherapy, HDR, interstitial, tandem and ovoid

## Abstract

**Purpose:**

This retrospective analysis was completed to investigate the use of a model‐based dose calculation algorithm (MBDCA) AcurosBV, for use in HDR BT treatments for locally advanced cervical cancer treated with tandem and ovoid applicators with interstitial needles.

**Methods:**

A cohort of 32 patients receiving post‐EBRT HDR brachytherapy boost with a prescription dose of 5.5 Gy × 5 fractions to the high‐risk clinical target volume (CTV_HR_) were selected for this study. For standard TG43 dose calculation, applicators were manually digitized on the planning images, while for AcurosBV calculations, solid renderings of Titanium Fletcher Suite Delclos (FSD) applicators included in BrachyVision were matched to those used clinically and Ti needles were manually digitized. The dose was recalculated using Varian's AcurosBV 13.5 and dose‐to‐medium‐in‐medium (D_m,m_) was reported. EQD2 values for targets and organs at risk were compared between dose calculation formalisms. D_90%_ and D_98%_ values were reported for the high and intermediate‐risk CTVs, and ${\;D}_{2\;cm^{3}}$ values were reported for OARs including bladder, rectum, sigmoid, bowel, and vagina. Due to variability within the patient cohort, the dosimetric impact of AcurosBV was investigated corresponding to planning image modality (CT vs. CBCT), presence of Ti needles, and contrast within vaginal balloons used to stabilize implants. AcurosBV showed lower dosimetric values for all plans compared to TG43.

**Results:**

The average ± standard deviation of dosimetric reduction in D_90%_ was 4.33 ± 0.09% for CTV_HR_ and 4.12 ± 0.09% for CTV_IR_. The reduction to OARs ${\;D}_{2\;cm^{3}}$ was: 4.99 ± 0.15% for bladder, 7.87 ± 0.16% for rectum, 5.79 ± 0.17% for sigmoid, 6.91 ± 0.14% for bowel, and 4.55 ± 0.14% for vagina.

**Conclusions:**

AcurosBV should be utilized for HDR BT GYN cases, treated with tandem and ovoid applicators, with high degrees of heterogeneity and calculated in tandem with TG43.

## 1 INTRODUCTION

Worldwide, cervical cancer is one of the most common malignancies amongst those with female anatomy.^1^ While there are a variety of treatment options, HDR brachytherapy in combination with an external beam is recommended for those with locally advanced disease.^2^, ^3^ Outcomes when brachytherapy is included in the treatment plan are more favorable than those without, and thus confidence in dose calculation algorithms is crucial to disease management. Traditionally, high dose rate brachytherapy (HDR BT) dose has been calculated using the American Association of Physicists in Medicine (AAPM) Task Group 43 (TG43) formalism which calculates the dose within the calculation volume as a homogeneous water environment.^4^ With this formalism, heterogeneities from tissues, applicators, air interfaces, etc. are not accounted for.

While TG43 formalism has been utilized for almost 30 years, there has been increased evidence that a model‐based dose calculation approach more accurately calculates dose by modeling heterogeneities within the calculation volume.^5^ Model‐based dose calculation algorithms (MBDCAs) use either hounsfield units (HU) from computed tomography (CT) or cone beam computed tomography (CBCT) images or physical material properties to model the calculation volume including all its complexities such as air interfaces, bone, and high “Z” materials (implants, applicators, etc.). MBDCAs model primary and secondary scatter interactions from the source by accounting for all heterogeneities present in the treatment volume.^6^ There are four MBDCAs available such as collapsed cone convolution, convolution superposition, grid based Boltzman solvers (GBBS), and Monte Carlo.^7^, ^8^

AcurosBV is a MBDCA of the GBBS type and was developed by Vassiliev et al. and later acquired and expanded on by Varian Medical Systems (VMS). AcurosBV models three distinct interactions from the source: photon to photon, photon to electron, and electron to electron. To account for all the material heterogeneity of the medium, AcurosBV uses macroscopic cross‐section data to assign each voxel with a range of density values to solve Linear Boltzman Transport Equation for each voxel. This is done by deriving mass density from HU taken from the planning CT image.^9^, ^10^ From these density values, the average density within one voxel is calculated, and it is automatically converted to material composition based upon the data from ICRP 23 (1975).^11^ These density values are not individually assigned to material but placed in density bins assigned across a range of material values. When solid applicators (SAs) are present within the calculation volume, the density is assigned based on the physical material included from the solid rendering in a library of SAs.^12^

AAPM Task Group 186 (TG186) recommends utilizing MBDCAs for brachytherapy in clinical applications.^5^ TG186 recommends two different dose reporting methods in MBDCA: $D_{w,\;m}$ and $D_{m,\;m}$. In $D_{x,\;y}$, *x* describes the reporting medium, and *y* describes the voxel characteristics used to calculate radiation transport. For example, TG43 formalism would report dose as $D_{w,\;w}$, dose‐to‐water‐in‐water. There is much discussion in the literature regarding which reporting scheme is most appropriate^13^, ^14^ and both have compelling evidence behind them. However, for HDR brachytherapy TG186 ultimately recommends the use of $D_{m,m}$.^5^

Various studies have been conducted on the dosimetric difference between MBDCA and standard approaches in both brachytherapy and external beam radiation therapy (EBRT). For GYN brachytherapy specifically, a review of the literature is provided in Table 1. All studies found that while there was a dosimetric reduction when using MBDCAs, the reduction was less than 5% and did not have a significant clinical impact. These studies, however, had small patient cohorts and concluded that further investigation was needed. More recently, Bi et al. completed a retrospective study of 30 patients undergoing HDR treatment using various types of HDR applicators for cervical cancer and compared their TG43 dose to that of an MBDCA. In the subset of patients receiving tandem and ovoid (T&O) HDR (*N* = 10), they found that the dose difference with these applicators was around 10% and recommended this approach for titanium applicators.^15^

**TABLE 1:** Literature review to report dose difference of ${\mathrm{CTV}}_{\mathrm{HR}}$ between TG 43 and MBDCA dose calculations for HDR brachytherapy of cervical cancer.

Authors	Algorithm	GYN applicators	Date of publication	$\mathrm{CT}V_{\mathrm{HR}}$ ($D_{90}$)	Dose reporting methodology	Number of patients
Hyer et al.	Acuros	Tandem & ovoid	2012	1.9 ± 0.7 %	$D_{w,m}$	5
Jacob et al.	ACE	Tandem & Ring	2017	2.7 ± 1.3 %	$D_{w,m}$	10
Hofbauer et al.	Acuros1.4.0	Tandem & Ring	2016	0.47 ± 0.33 %	$D_{w,m}$	9
Mikell et al.	Acuros1.3.1	Tandem & ovoid	2012	Pt A. < 5%	$D_{w,m}$	26

In this work, dosimetric differences between TG43 formalism and MBDCA (AcurosBV) brachytherapy dose calculation approaches were investigated specifically for locally advanced cervical cancer treated with T&O applicators with and without interstitial needles. The AcurosBV algorithm together with the SAs used were commissioned at our institution following the recommendations provided in TG186^5^.

Comparisons were made to examine dosimetric changes caused by various planning factors which included different planning imaging modality (CT vs. CBCT), dose reporting medium, presence of needles, and contrast within vaginal balloons. This work was done to help guide a change in clinical flow from utilizing TG43 only, to AcurosBV, or some combination based on the results.

## 2 METHODS & MATERIALS

### 2.1 Clinical workflow: Prescription, applicator placement, imaging, and treatment planning

Locally advanced cervical cancer patients treated between 2014 and 2018 were included in this Institutional Review Board (IRB) approved protocol. These patients were treated with 45 Gy over 25 fractions of EBRT to the whole pelvis. If gross nodal disease and parametrial involvement were present, those regions received an EBRT boosts to 60–70 Gy and 55–60 Gy, respectively. Following EBRT, the HDR boost (5.5 Gy × 5 fractions) used a hybrid (intracavitary/interstitial) approach with Varian's Titanium Fletcher Suite Delclos (FSD)‐style, magnetic resonance (MR) compatible, flexible geometry applicators (Varian part number—GM11006860) and additional 250 mm long titanium interstitial needles (Varian part number—GM11010230).

Alatus vaginal balloons (AngioDynamics, Latham, NY) were used to stabilize the FSD T&O applicator and push the bladder and rectum away from the target. Using transparent film dressing Tegaderm (3M, St. Paul, MN), the anterior vaginal balloon was secured to the left ovoid and the posterior vaginal balloon to the right ovoid. For the patients included in this study, the vaginal balloons were filled with an iodinated contrast (IsoVue‐300 or 370, Bracco Diagnostics, Princeton, NJ) and saline to a 5% and 95% ratio, respectively. Transabdominal ultrasound imaging (BK Focus500, Curved Array 8830 probe, BK Medical, Burlington MA) was used for tandem positioning, and the in‐suite imaging device (CT (1 mm slice thickness) or CBCT) was used for verification of needle position and depth throughout the insertion process.

To acquire treatment planning CT images, one of two different systems were used: Acuity CBCT (Varian Medical Systems, Palo Alto, CA) between 2014 and 2017 and Airo CT (BrainLab, Munich, Germany) starting 2018. The systems were used for applicator placement verification and applicator reconstruction for planning. Once the CT images were acquired, the patient was transferred to the MRI suite using a HoverMatt (HoverTech International, Allentown, PA) to limit patient movement. T1 and T2‐weighted MRI sequences were acquired on a 3T Siemens Magnetom Skyra (Siemens Healthineers, Malvern, PA). T2‐weighted imaging was used for tumor and Organ at Risk (OAR) delineation, and a high‐resolution isotropic acquisition (1 mm pixel size) T1‐weighted scan was acquired for applicator and needle visualization and co‐registration with the planning CT. Contours drawn on the T2‐weighted MR images include both high‐risk and intermediate‐risk CTVs, and bowel, bladder, sigmoid colon, rectum, and vagina (vaginal wall).

Treatment planning was performed using BrachyVision 15.5 (BV) and treatments were delivered using a GammaMedplus iX afterloader (VMS) using Ir‐192 with nominal source strength of 407 mGy m^2^/h. During treatment planning the applicators and needles were manually reconstructed on the CT images. The dwell time distribution was defined using a combination of standard dwell time weighting with normalization to Point A and manual optimization by a physician. The dwell positions were 5 mm apart. The dose was then calculated in BV using the TG43 formalism for a clinical treatment plan.^2^

The goal of this clinical protocol was to achieve an equivalent dose in 2 Gy fractions (EQD2) of 85 Gy or greater to ${\mathrm{CTV}}_{\mathrm{HR}}\;D_{90\%}$ from combined brachytherapy and EBRT. OAR $D_{2\;cm^{3}}$ dose limitations were 70 Gy for the rectum and sigmoid, and 75 Gy for the bladder.^16^ Our clinic also reports $D_{2\;cm^{3}}$ to vaginal mucosa with a EQD2 target of 110 Gy as a softer constraint.^16^ Note that *α*/*β* value used for EQD2 calculation is 10 for the target and 3 for OARs, respectively.^17^

### 2.2 Retrospective re‐planning

For the 32 patients in this study, a total of 156 brachytherapy boost fractions were re‐planned using a new flow adapted for the use of AcurosBV 13.5. Each clinical plan was copied, and the manually reconstructed T&O applicators were replaced with SA renderings, ensuring geometric coincidence of the dwell positions verified by plan comparison. Titanium needles remained unchanged. The dwell times in each applicator were kept the same as those in the clinical plan, dose was recalculated with AcurosBV 13.5 and the dose was reported as dose to medium‐in‐medium (D_m_,_m_). Rather than AcurosBV assigning material properties based on the HU values in the applicator regions from the images, it uses a predefined density value from the solid renderings, which overrides the underlying CT data within the applicator. Solid renderings of the titanium interstitial needles are not included in the SA library so material properties for the needles were based on the CT calibration curve and associated materials. AcurosBV assigns the density of the Ti needles as 4.42 $(\frac{g}{{\mathrm{cm}}^{3}}$).^12^

### 2.3 Statistical analysis

A quantitative comparison between the two dose calculations was performed using Bland‐Altman analysis (BAA).^18^ BAA calculates the mean difference and standard deviation between a reference or parent method (P) and a secondary method (Q) to set up limits of agreement. Typically, the reference method is a standard approach or well‐known technique. For this study TG43 calculations were considered as the reference method.^16^ The two plans were compared by evaluating the difference of EQD2 values using Equation (1), where $D_{V\;}$ is $D_{2\;cm^{3}}$ for OARs and $D_{90\%}$ for targets.

(1) $$\Delta D_{V(\mathrm{EQD}2)\;}=D_{V,\mathrm{TG}43\left(\mathrm{EQD}2\right)}-D_{V,\mathrm{AcurosBV}(\mathrm{EQD}2)}$$

Statistical significance was determined through statistical analyses including *t*‐testing, null hypothesis investigation, and Welch testing.^19^ It is considered statistically significant if the computed *p*‐value is <0.05. Statistical significance was evaluated using *t*‐testing, null hypothesis investigation, and Welch testing.

### 2.4 Effect of different planning image modalities: CT vs. CBCT

Due to the inclusion of both CBCT (70 plans) and CT (86 plans) image‐based plans, dosimetric differences between AcurosBV and TG43 formalism were also investigated between the two different imaging modalities. BrachyVision automatically correlates grayscale levels to HU values based on commissioning data which differs between CT modalities. The average change in EQD2 dose (Gy) and percent for both methods of imaging were compared as follows.

$$\begin{array}{c} \Delta D_{V,\mathrm{CT}\;}=D_{V,\mathrm{TG}43\left(\mathrm{CT}\right)}-D_{V,\mathrm{AcurosBV}\left(\mathrm{CT}\right)}\\ \Delta D_{V,\mathrm{CBCT}\;}={\;D}_{V,\mathrm{TG}43\left(\mathrm{CBCT}\right)}-{\;D}_{V,\mathrm{AcurosBV}\left(\mathrm{CBCT}\right)} \end{array}$$

Statistical significance was evaluated using the Welch test.^19^

### 2.5 Effect of using interstitial needles

A subset of implants (24 fractions) were treated exclusively with T&O applicators without the use of interstitial needles. This group of plans was compared to an equivalent number (24 fractions) of plans with a T&O applicator and 2–8 interstitial needles. The purpose of this analysis was to investigate if the presence of the needles influenced the overall dose distribution and, subsequently, the dosimetric change when using AcurosBV. Both sets of plans’ average dosimetric change (EQD2, Gy) and percent change were compared as in the previous section. The correlation between the two methods was investigated using BAA. Statistical significance was evaluated using *t*‐testing, null hypothesis investigation, and Welch testing.

### 2.6 Effect of using vaginal balloons with contrast

To investigate the dosimetric effect of IsoVue‐300 contrast within vaginal balloons, the balloon was contoured and assigned an HU value of zero for water/soft‐tissue equivalency. Twenty‐four plans were calculated with AcurosBV and compared to both the TG43 plan and the AcurosBV plan which included the original HU value found in the balloons. The two AcurosBV plan doses were then compared to the dose from the clinical plans as described previously. Average dosimetric change in EQD2 were reported. Statistical significance between contrast and no contrast plans was conducted by using Welch testing. The difference is considered statistically significant if the calculated *p*‐value is <0.05.

### 2.7 Effect of different dose reporting methods: D_w,m_ vs. D_m,m_

To compare results with previously published work (see Table 1) that reported $D_{w,m}$, a subset of 10 plans analyzed the dosimetric difference between the two dose reporting methods. The same statistical evaluation methods (*t*‐testing, null testing, and Welch tests) were used to compare the two different reporting methods.

## 3 RESULTS

This study includes 32 patients with a total of 156 fractions treated with T&O applicators with titanium interstitial needles. Table 2 shows the average EQD2 dosimetric change (Gy and percentage) for target structures and organs at risk. The AcurosBV calculated doses are always lower than TG43 calculated dose. The average dose reduction was 4.20 % for target and 5.90 % for OARs. The maximal dose reduction was 9.68% for target and 29.03% for vaginal wall OAR.

**TABLE 2:** CTV_HR_ & CTV_IR_ dosimetric difference (average ± standard deviation) and average dose from TG43 calculation. Dose difference is defined by (AcurosBV dose)—(TG43 dose). All doses are calculated as EQD2.

Target data			
${\mathrm{CTV}}_{\mathrm{HR}}{\;D}_{90\%}$	${\mathrm{CTV}}_{\mathrm{HR}}{\;D}_{98\%}$	${\mathrm{CTV}}_{\mathrm{IR}}{\;D}_{90\%}$	${\mathrm{CTV}}_{\mathrm{IR}}{\;D}_{98\%}$
(−0.41 ± 0.13) Gy	(−0.32 ± 0.12) Gy	(−0.21 ± 0.07) Gy	(−0.15 ± 0.06) Gy
(−4.33 ± 0.09) %	(−4.42 ± 0.12) %	(−4.12 ± 0.09) %	(−3.92 ± 0.12) %
Average TG43 Dose			
9.45 Gy	7.21 Gy	5.08 Gy	3.88 Gy

OAR data				
Bladder ${\;D}_{2\;cm^{3}}$	Bowel ${\;D}_{2\;cm^{3}}$	Sigmoid ${\;D}_{2\;cm^{3}}$	Rectum ${\;D}_{2\;cm^{3}}$	Vagina ${\;D}_{2\;cm^{3}}$
(−0.34 ± 0.16) Gy	(−0.24 ± 0.12) Gy	(−0.21 ± 0.10) Gy	(−0.20 ± 0.10) Gy	(−0.45 ± 0.26) Gy
(−5.05 ± 0.15) %	(−6.50 ± 0.22) %	(−5.78 ± 0.21) %	(−7.63 ± 0.21) %	(−4.56 ± 0.14) %
Average TG43 dose				
6.73 Gy	3.84 Gy	3.71 Gy	2.77 Gy	9.81 Gy

**FIGURE 1:**
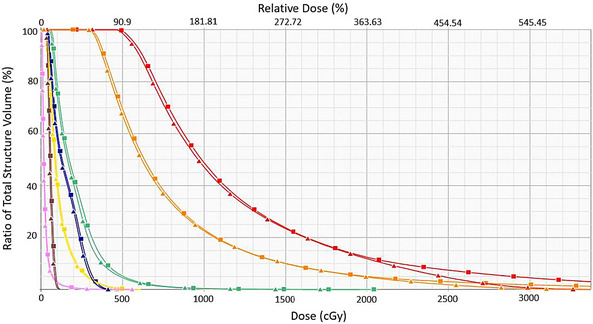
Axial (a/b) and sagittal (c/d) view of planning CT including isodose lines from TG43 (a/c) and AcurosBV (b/d) calculations. OAR and CTV structures shown are as follows vaginal wall (light green), bowel (pink), bladder (yellow), rectum (brown), sigmoid (blue), ${\mathrm{CTV}}_{\mathrm{HR}}$ (red), and ${\mathrm{CTV}}_{\mathrm{IR}}$ (orange).

**FIGURE 2:**
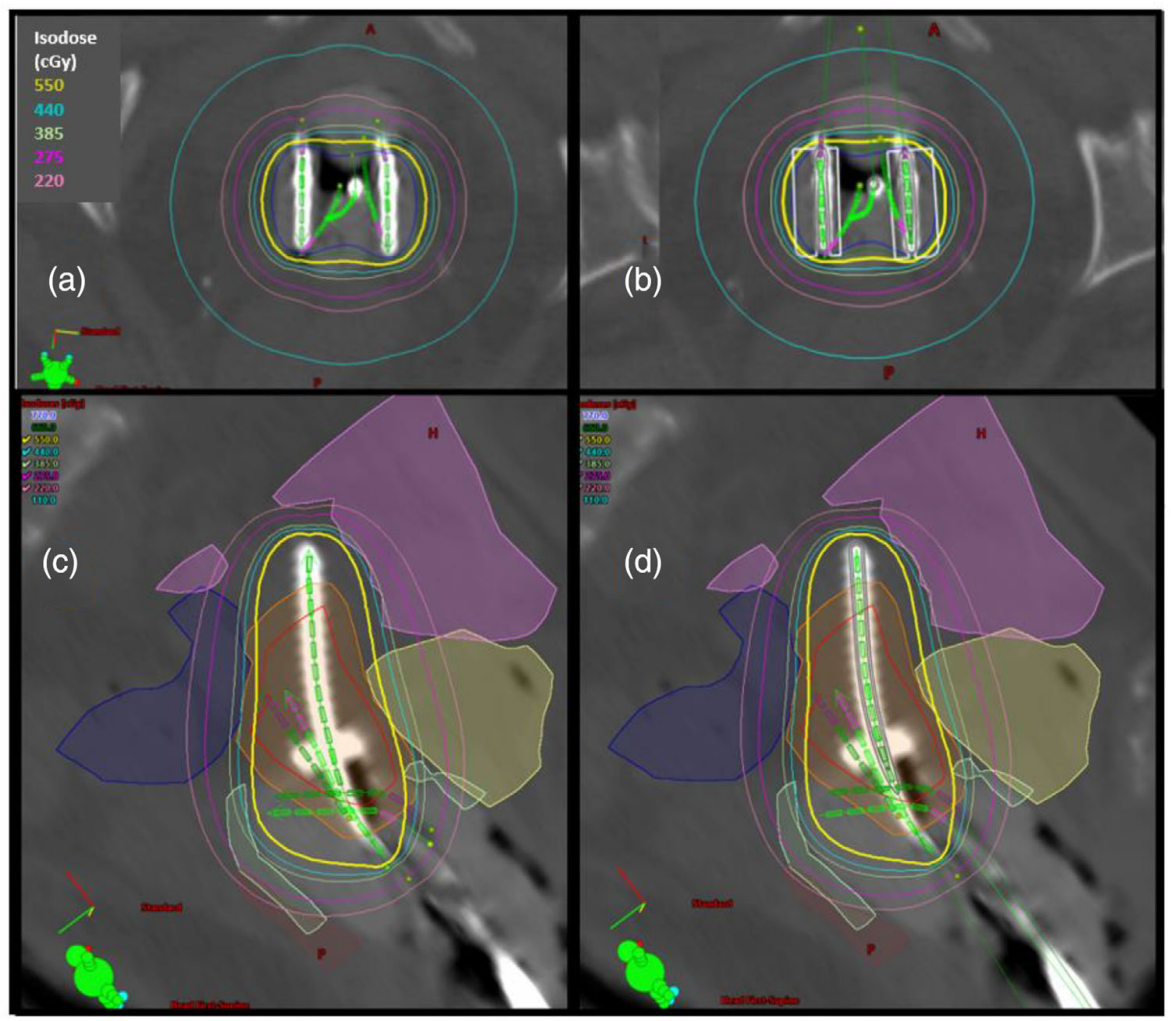
Cumulative DVH including TG43 (squares) and AcurosBV data (triangles). Vagina (light green), bowel (pink), bladder (yellow), rectum (brown), sigmoid (blue), ${\mathrm{CTV}}_{\mathrm{HR}}$ (red), and ${\mathrm{CTV}}_{\mathrm{IR}}$ (orange).

Figure 1 shows the planning CT images of a representative T&O fraction with target and OARs contours and physical isodose lines displayed. For this fraction, the cumulative DVH comparison is shown in Figure 2. The TG43 calculated ${\mathrm{CTV}}_{\mathrm{HR}}\;D_{90\%}$ dose was 8.09 Gy, and 7.70 Gy with AcurosBV (a dosimetric change of −0.39 Gy). The OARs showed a reduction to $D_{2\;cm^{3}}$ including vagina (−0.77 Gy), bowel (−0.51 Gy), and bladder (−0.43 Gy).

**FIGURE 3:**
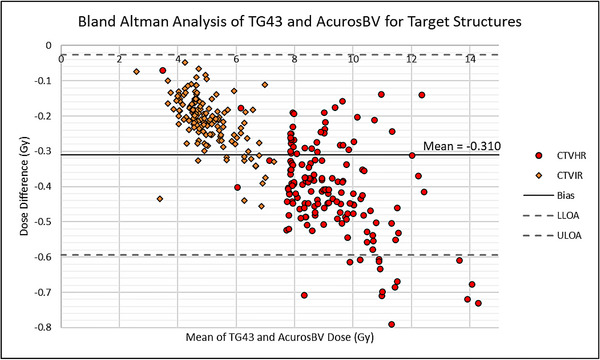
(a) Bland‐Altman plot of ${\mathrm{CTV}}_{\mathrm{HR}}$ (red circles) and ${\mathrm{CTV}}_{\mathrm{IR}}$ $D_{90\%}$ (orange diamonds) data for all fractions (*N* = 156). (b) Linear regression data for ${\mathrm{CTV}}_{\mathrm{HR}}$ (red circles) and ${\mathrm{CTV}}_{\mathrm{IR}}$ $D_{90\%}$ (orange diamonds).

**FIGURE 4:**
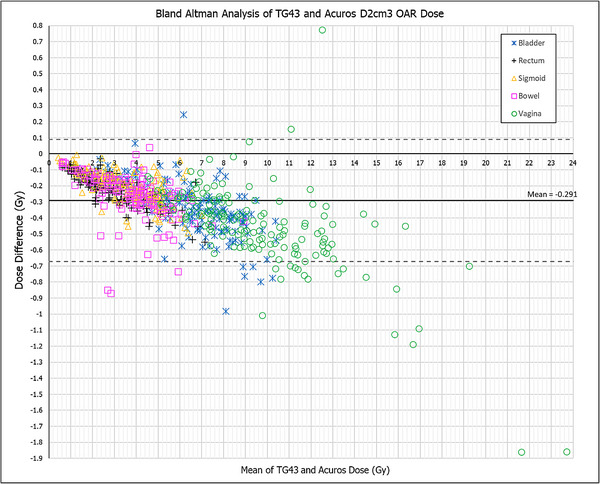
OAR Bland‐Altman analysis data for all fractions (*N* = 156).

The trend of lower dose from AcurosBV calculation can be seen in all the fractions with the negative differences (AcurosBV—TG43) for targets in the BAA plot Figure 3. Linear regression plots are also shown for ${\mathrm{CTV}}_{\mathrm{IR}}$ and ${\mathrm{CTV}}_{\mathrm{HR}}\;D_{90\%}$ are shown in Figure 3. The *p*‐value for each graph is <0.001 and the *r* value is equal to one indicating statistical significance and a strong positive correlation between the methods. OAR dose differences are shown in Figure 4. A similar trend to the CTV data is observed, however significantly more data falls outside of the two standard deviations. A few fractions do not correspond to the trend and lie above the zero‐line (*N* = 3). This indicates that the AcurosBV dose was higher than the TG43 dose.

### 3.1 Effect of different planning image modalities: CT vs. CBCT

When comparing the difference between dose calculation methods for specific imaging modalities (CT vs. CBCT), there is an insignificant statistical difference (*p*‐value of 0.24 calculated by the Welch test.) Table S1 summarizes TG43 versus AcurosBV doses for the use of different planning images.

### 3.2 Effect of using interstitial needles

When comparing the effects of AcurosBV calculation algorithm for T&O applicators (*N* = 24) versus T&O with interstitial needles (*N* = 24), there was no change in dose. Dose differences with T&O applicators was −0.42 ± 0.10 Gy (median dose difference ± standard deviation) and with T&O and needles was −0.42 ± 0.14 Gy.

### 3.3 Effect of using vaginal balloons with contrast

A subset of 24 fractions was selected and AcurosBV dose with and without contrast in vaginal balloons was investigated and TG43. The mean dose difference for ${\mathrm{CTV}}_{\mathrm{HR}}$ $D_{90\%}$ with contrast included in balloons was −0.397 ± 0.094 Gy. When the contrast value was set to being water equivalent, the dose difference was −0.429 ± 0.119 Gy.

### 3.4 Effect of using different dose reporting methods: $D_{w,m}$ and ${\;D}_{m,m}$

The choice of using D_m_,_m_ reporting methodology versus a D_w_,_m_ had little effect on the reduction using AcurosBV. On average, there was less than 1 cGy change between the reduction for the target structures for both $D_{90\%}$ and $\;D_{98\%}$. Mean dose difference for ${\mathrm{CTV}}_{\mathrm{HR}}$ $D_{90\%}$ reported using $D_{m,m}$ was −0.41 ± 0.13 Gy and reported as $D_{w,m}$ was −0.30 ± 0.15 Gy. Dose differences were statistically insignificant (*p*‐value of 0.61 calculated by the Welch test).

## 4 DISCUSSION

The study presented here represents the largest data set of its kind, comparing the dosimetric effect from the use of an MBDCA approach to TG43 for advanced cervical cancer patients in HDR BT. This is also the first to investigate if AcurosBV had any dosimetric impact based on planning images, contrast in vaginal balloons, and the presence of interstitial needles. While other studies have examined the difference between dose reporting methodology, this work indicates that differences are low enough that clinical significance is unlikely, specifically in HDR BT due to tissue density similarities within GYN volumes of interest. These tissues have mass‐energy absorption coefficients close to water within the energy range of Ir‐192. Bi et al. attributed the clinical significance of their work only to plans using Ti applicators because of the material composition of the applicators themselves.^15^ Their work showed that dosimetric differences with other applicators (Tandem and Ring (T&R) and cylinder) were negligible.

AcurosBV showed an average −4.42 ± 0.12% dosimetric reduction in high‐risk target volumes when compared to TG43 which is in good agreement with the current literature.^7^, ^20^, ^21^ Due to the deterministic nature of MBDCAs, AcurosBV can calculate the dose originating from scattered photons within the treatment volume by modeling the treatment medium and reporting the dose within the medium. Many previous studies in this area report the dose as D_w_,_m_, because this was the standard for early GBBS algorithms. AcurosBV now has the capability of reporting dose aligned with TG‐186′s recommendation as D_m_,_m_. Additionally, modeling the applicators as solid structures corrects for attenuation within the applicators themselves, leading to improved dosimetric accuracy. At this time, other studies examining dosimetric effects of an MBDCA approach on any disease site for HDR BT using CBCT versus CT imaging have not yet been conducted.

The average dosimetric reduction to ${\mathrm{CTV}}_{\mathrm{HR}}$ and ${\mathrm{CTV}}_{\mathrm{IR}}$ was −4.33 ± 0.09% (−0.41 ± 0.13 Gy) and −4.12 ± 0.09% (−0.21 ± 0.07 Gy), respectively. While this reduction is quite low, and may be clinically irrelevant, it could cause some cases to fall below the dose goal threshold for targets or OARs. In the 156‐fraction data set, seven fractions fell below the target metrics when planned with AcurosBV which was not the case when planned with TG43. This is an indication that a concurrent dose calculation with TG43 and AcurosBV may be a good solution for clinical implementation to ensure prescription doses are met when there is any question as to prescription dose coverage.

The dosimetric trends for OAR data are slightly more complex because, based on the organ, there is more variation outside of the standard deviations. Due to optimization and the nature of brachytherapy applicators being placed within CTVs, OARs receive smaller doses in comparison. An average dosimetric change of −0.2 to −0.3 Gy in OARs is a higher relative percentage than the same change in the ${\mathrm{CTV}}_{\mathrm{HR}}$. For example, a dose change of −0.15 Gy in OAR dose (3 Gy) is 5% while that in target dose (9 Gy) would be 1.67%. The vaginal wall shows the largest dose variation with an average TG43 dose of 9.81 Gy and a max dose of 24.7 Gy. This dose variation can be attributed to their proximity to the applicators themselves, but also to more variability and less accuracy in contouring. However, the average absolute dosimetric reduction with AcurosBV plans was around −0.4 Gy, similar to the reduction for the ${\mathrm{CTV}}_{\mathrm{HR}}$.

**FIGURE 5:**
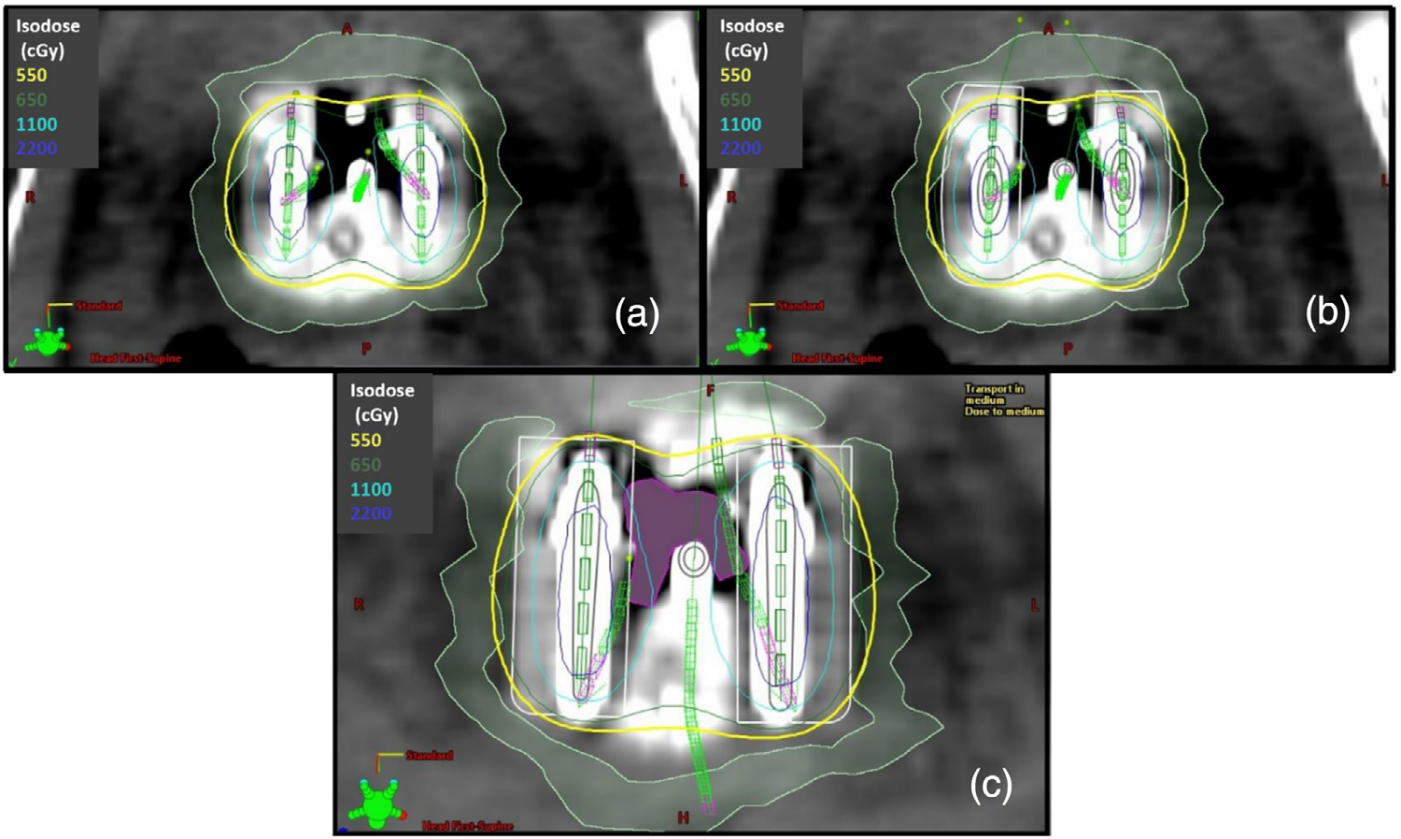
Axial slice of a patient planned with TG43 (a) and AcurosBV (b) showing changes in the isodose lines between the ovoids and vaginal wall contour (light green). (c) Air contour in between ovoids with AcurosBV calculated isodose.

These trends may point to another use for the AcurosBV approach in identification of hot spots based on anatomy or applicator placement. While this trend was found to be well observed across the data set as a whole, there are a few outliers specifically with the OAR analysis. Figure 5 shows axial planning images of one of these outliers with both TG43 (A) and AcurosBV (B) calculated dose distributions. AcurosBV accounts for differences in photon attenuation based on the material composition which was translated from the HU values of the planning CT or CBCT images. In Figure 5, air is present between the ovoids and this leads to an increase in dose to the vaginal walls in the lateral directions because photons are attenuated less in air than in water.^4^ In this case, the dose increased by 0.076 Gy when calculating with AcurosBV. To verify, the area of low density on the CT was contoured and made water equivalent (Figure 5c). Then, keeping all conditions the same, the dose was recalculated using AcurosBV and the dosimetric difference was found to be −0.42 Gy as opposed to 0.076 cGy with the air included.

Regarding the CBCT and CT study, further work is needed to evaluate this trend more thoroughly. AcurosBV, as described previously, assigns mass density values based on the HU from the planning images. Based on internal unpublished acceptance data for the CT and CBCT machines used in this study, HU values for various materials can vary between modalities. This could cause the AcurosBV density values to be inconsistent. However, at our institution, the CT numbers for materials used in QA and commissioning varied by less than 40 H. One other reason for the small dosimetric dependency could be that in the HDR BT energy range, Compton interactions dominate. Electron densities are also similar across the tissue in the pelvic region, and this would lead to small changes with AcurosBV.

The choice of dose reporting methodology has little effect on the dosimetric change when using AcurosBV over TG43. Within the HDR‐GYN treatment volume, most tissue can be assumed to have a density close to cartilage (1.06 $g/cm^{3}$), skeletal muscle (0.9693 $g/cm^{3}$), or adipose tissue ($0.92\;g/cm^{3})$.^12^, ^21^ AcurosBV uses the average material cross‐section density, calculated from the HU, to define the physical material in each voxel. The physical materials are assigned across a range of density values. For example, a voxel would be defined as “Air” if the average voxel density is anywhere between 0.0001 and 0.0204 $g/cm^{3}$. Large differences between material types, such as bone, or air, would lead to a larger dosimetric difference when calculated with AcurosBV. Cartilage, soft tissue, and adipose tissue would show less of a dosimetric change from water based on their density. This, coupled with the fact that absorbed dose to water and absorbed dose to tissue in the high‐energy (0.1–10 MeV) photon range are nearly equal,^5^ it is not surprising that these results indicate methods $D_{m,m}$ and $\;D_{w,m}$ are extremely similar.

In the case of the applicators themselves AcurosBV assigns the physical material a range of density values as described in the previous paragraph. For Ti applicators, the BrachyVision Reference Guide^12^ indicates that Ti is made up of three distinct elements, aluminum, titanium, and vanadium with weight fractions of 0.06, 0.9, and 0.04 respectively. This allows AcurosBV to define a material as Ti (density of 4.42 $g/cm^{3}$) if the material has an average voxel density of 3.56–6.21 $g/cm^{3}$. The plan images included high‐Z guide wires within the applicators for identification and visualization. The density of these wires and their effect on the assigned physical material was not studied. The HU value of these wires was very similar to the HU of the applicator themselves. It is reasonable, however, that, due to their small size, they would cause very little difference in dose calculated with AcurosBV because of a small amount of attenuation.

Clinically, there is potential for implementing AcurosBV as an end‐point dose calculation during the planning process. Due to the increased computational need for a MBDCA, plans would be created using TG43 dose calculation and iterated until an appropriate dose was given to target structures and constraints for normal tissue were met. It is noted that the current dosimetric goals in the clinical practice were determined by the previous clinical outcome data correlated with the dosimetric data calculated using TG43 formalism, not MBDCAs. Once a favorable dose coverage is achieved, AcurosBV dose would be calculated. However, AcurosBV takes additional time to calculate and more computation power which precludes the use of the dose‐shaping tool available in modern planning systems. Both the TG43 plan and the AcurosBV plans will be compared and inspected for large dosimetric changes. If warranted, the plan could be further manipulated at this time or continued through the clinical approval process. Any change to the clinical flow may require additional training for all faculty and staff involved in the planning process specifically so that everyone understands the differences between a dose to water, and a dose to medium reporting structure.

## 5 CONCLUSION

Clinical implementation of a MBDCA AcurosBV style algorithm is challenging due to both logistical complexities and strain on computational resources. The results of this study do not present enough clinical dosimetric change to warrant using an MBDCA approach for the primary algorithm for HDR ^192^Ir T&O gynecological treatments. However, MBDCA approach could be utilized as a supplemental check or verification tool. Due to calculation speed, TG43 could be utilized as the primary algorithm with AcurosBV calculated dose as a second verification check. It is our recommendation from this work, in agreement with the American Brachytherapy Society,^5^ that centers having commissioned AcurosBV start reporting their results to gain a repository of clinical data to support a transition to MBDCA primary calculations.

## AUTHOR CONTRIBUTIONS

Billie Ann Radcliffe, MS, carried out the retrospective treatment planning and all data acquisition for the study and is the primary author of the manuscript. Oana Craciunescu, PhD and Sheridan Meltsner, PhD, designed the study and assisted with data analysis and interpretation. Junzo Chino, MD, PhD, Diandra N. Ayala‐Peacock, MD, PhD, and Sarah J. Stephens, MD, determined patient cohort and protocol. Yongbok Kim, PhD, and all other authors contributed extensively to data analysis, interpretation, and editing of the final manuscript.

## CONFLICT OF INTEREST STATEMENT

The authors declare no conflicts of interest.

## Supporting information



Supporting Information
