# Supplementary material for: Retrospective assessment of HDR brachytherapy dose calculation methods in locally advanced cervical cancer patients: AcurosBV vs. AAPM TG43 formalism
Source: J Appl Clin Med Phys. 2024 Oct 9;26(1):e14549. doi: 10.1002/acm2.14549 (PMC11712453; doi:10.1002/acm2.14549)
Supplement: Supplementary file 1 — Supporting Information [file ACM2-26-e14549-s001.docx]

Supplemental Materials

Table 1. Dosimetric change data for plans based on CBCT and CT images.

| Image Type | $\text{CTV}_{\text{HR}}\text{ D}_{\text{90\%}}$ | $\text{CTV}_{\text{HR}}\text{ D}_{\text{98\%}}$ | $\text{CTV}_{\text{IR}}\text{ D}_{\text{90\%}}$ | $\text{CTV}_{\text{IR}}\text{ D}_{\text{98\%}}$ |
| --- | --- | --- | --- | --- |
| CT | - 0.39 ± 0.14 Gy | - 0.32 ± 0.12 Gy | - 0.20 ± 0.13 Gy | - 0.15 ± 0.07 Gy |
| CBCT | - 0.44 ± 0.11 Gy | - 0.35 ± 0.12 Gy | - 0.21 ± 0.14 Gy | - 0.16 ± 0.06 Gy |
| Average TG43 Dose: CT | 9.55 Gy | 7.36 Gy | 5.19 Gy | 3.97 Gy |
| Average TG43 Dose: CBCT | 9.32 Gy | 7.24 Gy | 4.97 Gy | 3.88 Gy |
